# Innovation in identifying metabolites from complex metabolome—Highlights of recent analytical platforms and protocols

**DOI:** 10.3389/fchem.2023.1129717

**Published:** 2023-01-25

**Authors:** Shi Qiu, Sifan Guo, Qiang Yang, Yiqiang Xie, Songqi Tang, Aihua Zhang

**Affiliations:** ^1^ International Advanced Functional Omics Platform, Scientific Experiment Center, Hainan Medical University, Haikou, China; ^2^ Graduate School, Heilongjiang University of Chinese Medicine, Harbin, China

**Keywords:** metabolites, functional biomarkers, metabolic pathway, metabolomics, diagnosis

## Abstract

Metabolites are closely intertwined genotypes that can provide clear information about the final phenotype. The high-throughput analysis platform used to identify candidate metabolites and describe their contributions can help to quickly detect metabolic characteristics from large spectral data, which may lead to peak data preprocessing, statistical analysis and functional interpretation. Developing a comprehensive strategy for discovering and verifying bioactive metabolites can provide a large number of new functional biomarkers, and then more closely reveal their functional changes, which has relevant biological significance for disease diagnosis and prognosis treatment.

In 2022, five studies on high-throughput mass spectrometry metabolomics analytical platforms and softwares for identifying metabolites and delineating their contribution were published in Nature Protocols (Fu et al., 2022; Horvath et al., 2022; Kilgour et al., 2022; Kirkwood et al., 2022; Pang et al., 2022). These studies have shown metabolic profiling of candidate metabolites as a dominant technology that enables rapid detection of the metabolic features in the spectral data, which may be followed by spectral and peak data preprocessing, statistical analysis and functional interpretation. These five publications facilitated the extraction of chemical signals, contributed to identification of novel metabolites and differential metabolites, and discovered new functional biomarkers related metabolites leading to better diagnosis, prognosis and treatment.

Metabolites are the final downstream products of protein translation and gene transcription or cellular perturbations to the proteome, genome or transcriptome, which serve as potential key links between genotype and environment and can provide a clear image of the final phenotype. Small-molecule metabolites can provide insights into unique metabolic mechanisms and therapeutic targets of diseases, and lead to personalized applications of metabolic phenotypes. Furthermore, metabolite identification or metabolic pathway changes will help to understand the pathophysiology of the disease and assist in determining therapeutic targets. Metabolomes are comprehensively characterized by measuring the small metabolites in cells, biofluids, organs, or other biological systems, and represents the upstream input from the environment and the downstream output from the genome. In particular, due to the huge complexity of metabolome, there is urgency to developed various powerful tools to facilitate spectral data processing and interpretation that help to identify bioactive metabolites. Identification of small molecule metabolite is one of the most important processes in the discovery stage and can be performed by a range of established innovative technologies (Figure 1). Therefore, it is highly demanded to expand metabolites coverage, and optimize workflow by assessing multiple metabolic signals as possible to understand the biological role of metabolites. This minireview provides a brief overview and highlights of these five recent articles.

**FIGURE 1:**
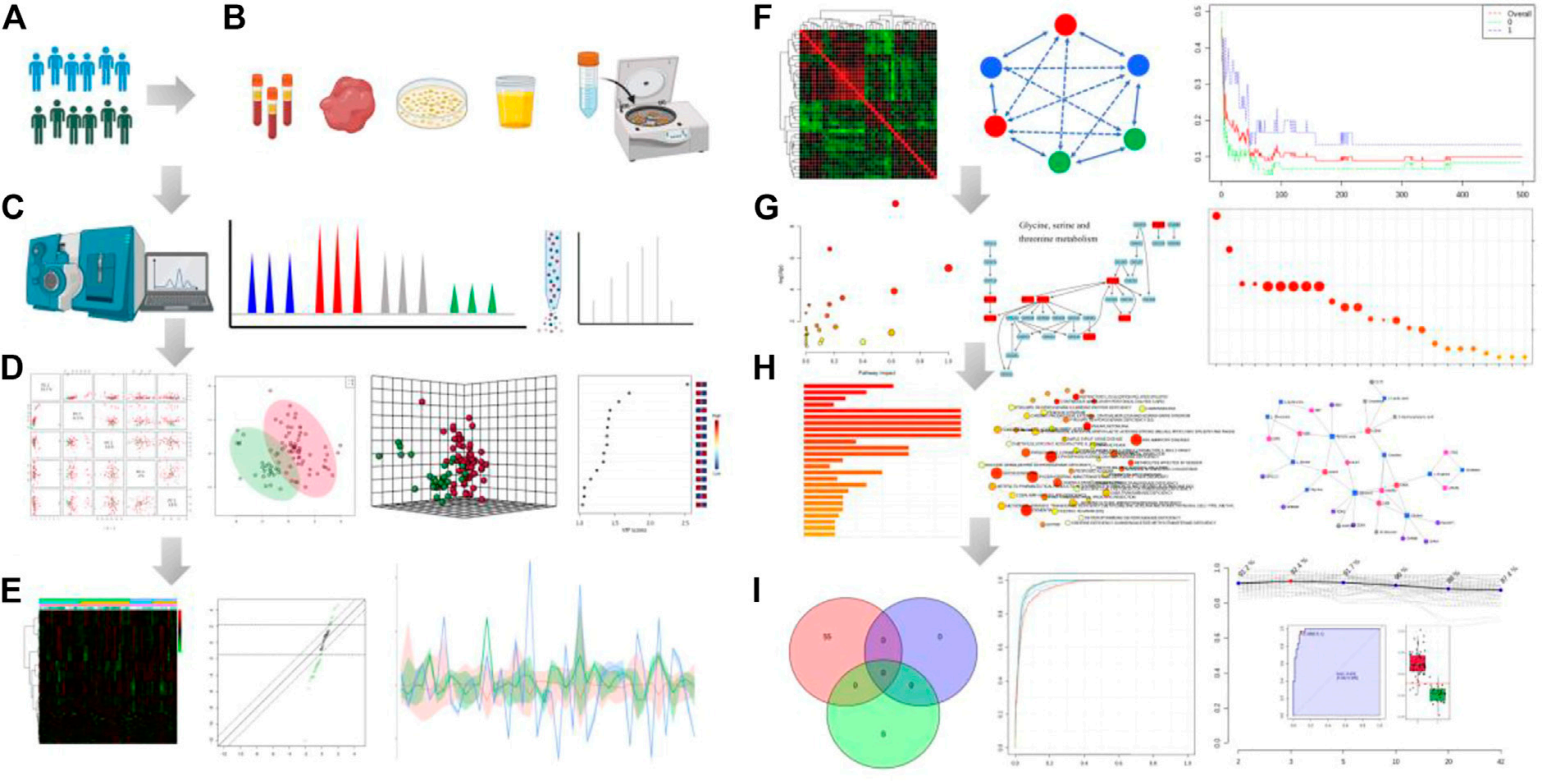
Analytical workflow of typical metabolomic analysis. It includes several basic steps: experimental design **(A)**, sample collection **(B)**, metabolite profling **(C)**, data analysis **(D−F)**, functional interpretation **(G, H)** and potential application of the integrated datasets **(I)**. Step **(A)** The experimental design based on phenotype analysis or diagnosis and treatment. Step **(B)** Sample preparation through deproteinization and/or centrifugation of biofluids. Step **(C)** Metabolite separation on a column (chromatography) and detection of analyte signal through MS or NMR spectroscopy. Metabolites can be identified on the basis of a combination of retention time and MS signature. Step **(D)** The data pre-processing and normalization of raw signals). Then, pattern recognition analysis and computational methods after data collection. Step **(E)** Expression analysis of the differential metabolites by which data is filtered for significant biomarkers of interest. Heatmap plot shows the differential metabolites in the statistical analysis function. Step **(F)** Clustering correlation patterns analysis among different data sets. Step **(G)** Pathway enrichment overview. Circle size and color are based on the pathway size and *p*-value. Step **(H)** The enriched metabolism pathway and joint pathway analysis in the correlation network. Step **(I)** Analysis model of diagnosis, prognosis and treatment based on the candidate metabolite features using classical univariate and multivariate ROC curve analyses. All images were obtained using the example data provided by the MetaboAnalyst 5.0 and figures also created by BioRender.

Recently, a study published in *Nature Protocols* by Horvath *et al.* developed a LC/MS quantitative analytical platform for liquid chromatography-tandem mass spectrometry metabolomics method for targeting analysis and monitoring the content change of microbially derived short-chain fatty acids and neurotransmitters from the bacterial cultures, organoid cultures and *in vivo* samples (Horvath et al., 2022). This method has been customized for the targeted metabolite measurements of different microbial subsets and growth media. It can comprehensively specifically identify microbial signals within the host, and evaluate the relationship between microbial colonization and neurotransmitters of gut-brain axis *in vivo* and *in vitro* (media and organoids) models, reveal the contributions of microbes to host neurotransmitters, and dissect the important bidirectional connections, between commensal microbes and host. In this study, the authors demonstrated a systematic approach to reveal how the bacteria regulate the CNS and gut, provide valuable information by determining the specific pathways into the functional mechanisms that specific microbes influence host metabolism (Horvath et al., 2022). In regard to cellular metabolism, Kilgour et al., focused on performing identification of general metabolic characteristics for specific cell populations by high-dimensional flow cytometry and MS-based metabolite profiling. They shared the general workflow of biological sample collection and storage (i.e., blood, tumor, and ascites) and cell enrichment analysis to uncover the unique metabolic difference in the tumor microenvironment (Kilgour et al., 2022). Furthermore, it can be applied to reveal credible information on the bioindicators during cellular processes and functional metabolic mechanisms in different cell types under extensive experimental conditions.

Kirkwood and colleagues have analyzed lipidome data by the open-source software *Skyline* tool to improve the capabilities of lipid characterization and annotation beyond traditional mass spectrometry (MS)-based approaches (Kirkwood et al., 2022). Importantly, they expanded capabilities of lipid molecule discovery, identification, quantification and annotation validation by integrating multidimensional liquid chromatography, mass spectrometry, ion mobility spectrometry, collision-induced dissociation and library editing. Pang et al., have used the online *MetaboAnalyst* platform (www.metaboanalyst.ca) to conduct high-resolution mass spectrometry spectra processing, statistical analysis of complex metabolic datasets, and joint-pathway analysis of multi-omics integration (Pang et al., 2022). It can provide a largely automated workflow for functional interpretation and analysis to identify critical features through efficient optimization of spectra processing parameters by default settings. This platform allocates small metabolites to biological pathways and enhances sensitivity and versatility of metabolites, precisely facilitates exploration of overall metabolic changes that related to disease alteration, from whole organisms to single cells, and offers invaluable insights into the metabolic function and targets. Fu et al., have applied *NOREVA* to facilitate optimizing the multiclass metabolomic data processing (Fu et al., 2022). This protocol includes data filtering and imputation, correction, transformation, normalization and performance assessment. Open source code is fully provided in this protocol, allowing users to customize and improve NOREVA functions. Interestingly, according to the size of the analysis data, the execution time can vary from a few minutes to several hours.

Taken together, these helpful studies provide the technical advances for identifying active metabolites and greatly broaden the coverage level of metabolites for exploring metabolic altered dimensions of diseases. The described analytical platforms and software tools provide some advantages in the sensitivity, dynamic range, reproducibility, time-saving and throughput for the combination of qualitative and quantitative. Rapid analysis of complex metabolome requires the specialized tools for the preprocessing raw data followed by multivariate statistical analysis, omics data mining and bioinformatics integration and functional interpretation. In this setting, *MetaboAnalyst* includes numerous modules for metabolite enrichment and relevant metabolic pathway analysis with pathophysiological mechanism by establishing the network interaction and visualization map (Pang et al., 2022).

Despite these tremendous advances, at present, no single analytical method can fulfill the mission of identification of entire metabolome in the large sample sizes. Future works aimed at developing the integrated strategy for enhancing the accuracy of discovering a list of biologically active metabolites with pathological basis of diseases, could provide a large number of the relatively abundant ions and acquire more reliable identifications, and then shed light on the functional changes of small molecule metabolites more closely. Moreover, it should establish multi-cycle training optimization models of experimental and clinical studies to validate the overall prediction performance of particular metabolite bioactivity and relevant biological significance for disease diagnosis and prognostic treatment that are worthy of further exploration. In the future, the integrated use of computational algorithms, artificial intelligence, and big data mining method will improve the precise identification ability of metabolites for resulting optimization and verification.
